# Antibiotics Use among Patients with Acute Exacerbation of Chronic Obstructive Pulmonary Disease in the Department of Internal Medicine of a Tertiary Care Centre: A Descriptive Cross-sectional Study

**DOI:** 10.31729/jnma.7512

**Published:** 2022-06-30

**Authors:** Ushab Rana Mohsin, Nabin Simkhada, Bishnu Deep Pathak, Bishal Dhakal, Binaya Subedi, Dilip Thapa, Bishnu Prasad Shrestha, Om Prakash Tandon, Sanjaya Shrestha, Shriya Sharma, Aakriti Adhikari

**Affiliations:** 1 Department of Pulmonology, Shree Birendra Hospital, Chhauni, Kathmandu, Nepal; 2 Department of Internal Medicine, Shree Birendra Hospital, Chhauni, Kathmandu, Nepal; 3 Shree Birendra Hospital, Chhauni, Kathmandu, Nepal

**Keywords:** *anti-bacterial agents*, *chronic obstructive pulmonary disease*, *guideline adherence*

## Abstract

**Introduction:**

Acute exacerbation of chronic obstructive pulmonary disease is a life-threatening condition triggered by infections or non-infectious agents. Antibiotics use in such cases prevents severe deterioration and treatment failure. Past studies have shown inappropriate use of antibiotics in different health care settings. The objective of this study was to find out the prevalence of antibiotics use in patients with acute exacerbation of chronic obstructive pulmonary disease in the Department of Internal Medicine of a tertiary care centre.

**Methods:**

A descriptive cross-sectional study was conducted among 108 patients with acute exacerbation of Chronic Obstructive Pulmonary Disease admitted to Department of Internal Medicine of a tertiary care centre from 12^th^ February, 2022 to 15^th^ April, 2022 after taking ethical clearance from Institutional Review Committee (Reference number: 417). Convenience sampling was done. Data analysis was done using the Statistical Package for the Social Sciences version 23.0. Point estimate at 95% Confidence Interval was calculated along with frequency and percentage for binary data along with median and interquartile range for continuous data.

**Results:**

The prevalence of antibiotics use among study participants was 106 (98.15%) (95.61-100.69 at a 95% Confidence Interval). Penicillin 82 (75.93%) was the most commonly used antibiotics group.

**Conclusions:**

The use of antibiotics in acute exacerbation of chronic obstructive pulmonary disease was higher as compared to other similar studies.

## INTRODUCTION

Acute Exacerbation of Chronic Obstructive Pulmonary Disease (AECOPD) is a life-threatening event characterised by worsening of a patient's baseline symptoms like dyspnea, cough and sputum production. It is the fourth leading cause of death worldwide.^1,2^ The exacerbations may be triggered by infectious or non-infectious agents.^3,4^

The antibiotics in Chronic Obstructive Pulmonary Disease (COPD) exacerbation prevent severe deterioration, shorten recovery time, and decrease the risk of treatment failure and early relapse.^5^ However, there is no sufficient evidence for their routine clinical use. Past studies have shown inappropriate use of antibiotics in different health care settings.^4^ Till date, there are very few studies done on antibiotic use in AECOPD in our setting.

The objective of this study was to find out the prevalence of use of antibiotics in patients with acute exacerbation of chronic obstructive pulmonary disease in the Department of Internal Medicine of a tertiary care centre.

## METHODS

This descriptive cross-sectional study was conducted in the Department of Pulmonology of a tertiary care centre, Kathmandu, Nepal for a duration of 3 months from 12^th^ February, 2022 to 15^th^ April, 2022. The ethical approval was taken from the Institutional Review Committee (IRC), Nepalese Army Institute of Health Sciences, Bhandarkhal, Kathmandu (Reference number: 417). All the patients with AECOPD admitted to the respiratory ward were included in the study. The patients under 18 years of age, with concomitant asthma, and those who tested Real-time Polymerase Chain reaction (RT-PCR) positive for Coronavirus Disease-19 (COVID-19) were excluded from our study. Convenience sampling was done. The sample size was calculated using the following formula:


$$\begin{array}{ll} \text{n} & ={\text{Z}}^{2}\times \frac{\text{p}\times \text{q}}{{\text{e}}^{\text{2}}}\\ & =1.96^{2}\times \frac{0.86\times 0.14}{0.07^{2}}\\ & =94 \end{array}$$

Where,

n = minimum required sample size
Z = 1.96 at 95% Confidence Interval (CI)
p = prevalence of antibiotics use in AECOPD, 86%^6^
q = 1-p
e = margin of error, 7%

Considering a non-response rate of 10%, the final sample size was 103. However, we took 108 cases for our study. The required data were collected after taking informed verbal consent from the cases admitted to the respiratory ward with the primary diagnosis of acute exacerbation of COPD. It included sociodemographic characteristics, comorbidities, baseline COPD profile (duration since diagnosis, regular medications, past exacerbations), smoking status, presenting complaints, antibiotics prescription, and outcomes (mortality, complications, intensive care unit admission, mechanical ventilation and duration of hospital stay).

The concordance of antibiotic prescription with the Global Initiative for Chronic Obstructive Lung Disease (GOLD) 2021 was studied. The guidelines recommend the use of antibiotics for those who have three cardinal symptoms: increase in dyspnoea, sputum volume and purulence; have two of the cardinal symptoms if increased purulence of sputum in one of these; or require mechanical ventilation (invasive or noninvasive). The recommended duration of therapy is five to seven days, and the choice of antibiotics is based on local bacterial resistance patterns.^5^

The data analysis was done using the Statistical Package for the Social Sciences (SPSS) version 23.0. Point estimate at 95% Confidence Interval was calculated along with frequency and proportion for binary data along with median and interquartile range for continuous data.

## RESULTS

Of the total of 108 patients with acute exacerbation of COPD, the prevalence of antibiotics use was 106 (98.15%) (95.61-100.69 at 95% Confidence Interval). The most commonly used antibiotics group was penicillin 82 (75.93%), followed by macrolide 64 (59.26%) and fluoroquinolones 14 (12.96%) (Table 1).

**Table 1:** Antibiotics prescription (n = 106)

Variables	n (%)
**Antibiotics prescribed**	106 (98.15)
**Concordance with GOLD-2019 guidelines**	58 (53.70)
**Antibiotics groups**	
Penicillin	82 (75.93)
Macrolide	64 (59.26)
Fluoroquinolone	14 (12.96)
Cephalosporin	13 (12.04)
Aminoglycoside	4 (3.70)
Meropenem	3 (2.78)

The overall median duration of antibiotics use was 5 (5-7) days. Out of these, the average duration of oral and injectable antibiotics were 5 (5-5) days and 5 (37) days respectively. Fifty-eight (53.70%) out of 106 antibiotics prescriptions were concordant with GOLD-2021 guidelines. The rest of the 48 (46.30%) cases did not meet the prescription guidelines. Out of the cases treated with antibiotics, 34 (32.08%) were males and 72 (67.92%) were females. The mean age was 72.22±9.10 years. Most of them 76 (71.70%) belonged to a joint family (Table 2).

**Table 2:** Socio-demographic and clinical characteristics of AECOPD patients (n = 106).

Variables	n (%)
**Gender**	
Male	34 (32.08)
Female	72 (67.92)
**Family**	
Nuclear	30 (28.30)
Joint	76 (71.70)
**Taking medications for COPD**	
Yes	102 (96.23)
No	4 (3.77)
**Domiciliary oxygen**	
Yes	53 (50.00)
No	53 (50.00)
**Comorbidities**	
Hypertension only	33 (31.13)
Diabetes mellitus only	5 (4.72)
Both hypertension and diabetes mellitus	19 (17.92)
Others	13 (12.26)
None	36 (33.96)
**Smoking status**	
Current smoker	17 (16.04)
Past smoker	79 (74.53)
No smoking	10 (9.43)
**Cough**	
Productive	30 (28.30)
Non-productive	9 (8.49)
No	67 (63.21)
**Increase in sputum volume**	
Yes	29 (27.36)
No	77 (72.64)
**Increase in sputum purulence**	
Yes	27 (25.47)
No	79 (74.53)
**Fever**	
Yes	13 (12.26)
No	93 (87.74)
**Chest pain**	
Yes	12 (11.32)
No	94 (88.68)
**Bilateral swelling of legs**	
Yes	16 (15.09)
No	90 (84.91)
**Bilateral crepitations**	
Yes	63 (59.43)
No	43 (40.57)
**Bilateral wheeze**	
Yes	60 (56.60)
No	46 (43.40)

The average duration of COPD in antibiotic-treated cases was 6 (3-12) years. One hundred and two (96.23%) patients were taking medications for COPD, which mainly included inhalational steroids. Four (3.77%) of them were not taking any medication. Fifty-three (50.00%) patients were on domiciliary oxygen therapy. On average, they had 2 (1-4) past exacerbations as well as hospitalizations due to them.

Regarding co-morbidities, 33 (31.13%) patients had hypertension only, whereas 19 (17.92%) had both hypertension and diabetes mellitus. Thirteen (12.26%) patients had other comorbidities like Coronary Artery Disease (CAD), Chronic Kidney Disease (CKD), stroke, hypothyroidism and psychiatric disorders. Majority of the cases 79 (74.53%) were past smokers. Seventeen (16.04%) were current smokers, and the remaining 10 (9.43%) had never smoked. The average smoking pack-year was 15 (7-30).

All 106 (100%) patients presented with complaints of shortness of breath. Thirty-nine (36.79%) cases had cough, with 30 (28.30%) of them reporting production of sputum. However, only 29 (27.36%) and 27 (25.47%) reported an increase in sputum volume and purulence respectively. Likewise, fever, chest pain and bilateral swelling of legs were present at admission in 13 (12.26%), 12 (11.32%) and 16 (15.09%) cases respectively. On clinical examination at presentation, 63 (59.43%) had bilateral crepitations and 60 (56.60%) had wheeze.

Among COPD exacerbation cases who were treated with antibiotics, 6 (5.66%) died during the course of their hospital stay (Table 3).

**Table 3:** Outcomes of AECOPD (n= 106).

Outcomes	n (%)
**Mortality**	
Yes	6 (5.66)
No	100 (94.34)
**Mechanical ventilation**	
Yes	49 (46.23)
No	57 (53.77)
**Complications**	
Yes	59 (55.66)
No	47 (44.34)
**Complications**	
Cor pulmonale	20 (18.87)
Pneumonia	16 (15.09)
Respiratory failure	17 (16.04)
Sepsis	3 (2.83)
Shock	2 (1.89)
Pleural effusion	1 (0.94)
**ICU admission**	
Yes	32 (30.19)
No	74 (69.81)

Fifty-nine (55.66%) suffered at least one complication, the most common being cor-pulmonale in 20 (18.87%), followed by respiratory failure in 17 (16.04%) and pneumonia in 16 (15.09%). Thirty-two (30.19%) cases required Intensive Care Unit (ICU) admission. Forty-nine (46.23%) patients required mechanical ventilation, which included Bi-level Positive Airway Pressure (BiPAP) and endotracheal intubation. The overall median duration of hospital stay was 6 (5-8) days.

## DISCUSSION

COPD is a progressive disorder with significant morbidity and mortality. On average, a Chronic Obstructive Pulmonary Disease (COPD) patient gets one to three exacerbations per year.^7^ About 30% of exacerbations are infectious, and half of these are of bacterial origin.^4^ Co-infection of viruses and bacteria has been detected in 25% of the cases.^8^ Frequent exacerbations deteriorate lung function, diminish the quality of life, and increase the risk of death.^9^ Antibiotics in COPD exacerbation are found to speed up recovery and prevent treatment failure.^5^ However, their use without meeting criteria may cause polypharmacy, increased cost factors, unwanted side effects, and microbial resistance.^10,11^ Prescribing antibiotics for exacerbations has remained a controversy till now. Mild exacerbations are often non-bacterial and do not require antibiotic treatment.

In our study, 106 out of 108 (98.15%) cases with AECOPD received antibiotics. This proportion was higher in contrast to studies done in other centres.^6,12,13^ However, this finding was almost identical to another study on a similar topic conducted in another tertiary hospital in Nepal.^14^ In our study, 53.70% of the cases treated with antibiotics met G0LD-2021 guidelines for antibiotics in AECOPD. This was a significant finding in our study where more than half of the cases were receiving antibiotics as per the standard guidelines. And, it was slightly lower than that reported by the other two studies.^6,15^ G0LD-2021 guidelines recommend the use of antibiotics for those who have three cardinal symptoms: increase in dyspnea, sputum volume and purulence; have two of the cardinal symptoms if increased purulence of sputum in one of these; or require mechanical ventilation (invasive or non-invasive). The duration of therapy is five to seven days, and the choice of antibiotics is based on local bacterial resistance patterns. Empirically, aminopenicillin with clavulanic acid, Macrolides or Tetracycline can be prescribed.^5^

In the present study, all the patients presented with an increase in shortness of breath. However, an increase in sputum volume and purulence was present in 27.36% and 25.47% of patients respectively. This was consistent with a study where an increase in sputum volume and purulence were present in only minority of the cases like ours.^12^ But in contrast to this, in a study from Switzerland, sputum volume and purulence were found in majority of cases with COPD exacerbation.^16^

The commonly used antibiotics in our setting were penicillin (75.93%) followed by macrolides (59.26%) and fluoroquinolones (12.96%). Similar findings were reported by the studies conducted in similar settings for AECOPD.^12,13,16^ However, the Penicillin group of antibiotics was least used in two settings.^15,17^ The Macrolides and Cephalosporin groups were among the antibiotics to be used as an alternative to penicillin in most of the studies.^15,17,18^ Based on the GOLD-2021 and European Respiratory Society (ERS) guidelines, Co-amoxiclav is the preferred empirical antibiotic for AECOPD.^19^ However, the preference of antibiotics has been based on the local resistance pattern and physician expertise and experience.^5,19^ The choice of antibiotics in AECOPD is still in controversy.

The total median duration of oral and injectable antibiotics was five days each in our setting. This was in concordance with the GOLD-2021 guidelines, and similar to a study mentioned above. However, the median duration for intravenous antibiotics was three days in the latter study.^12^ Although there are discrepancies regarding the use of antibiotics in AECOPD, a large study has demonstrated the superiority of antibiotics over placebo based on the category of exacerbations.^20^

In regards to outcomes of AECOPD, only 5.66% died during hospital stay who were treated with antibiotics. This was a similar outcome as reported in a study on the impact of antibiotics in AECOPD.^21^ Likewise, 30.19% of cases required ICU admission, which was low as compared to simple ward management (69.81%). This also shows the benefit of antibiotics in AECOPD to lower the rates of ICU admission. However, over half of the cases (55.66%) had at least one complication. The most common being cor-pulmonale (18.87%) followed by respiratory failure (16.04%) and pneumonia (15.09%). The need for mechanical ventilation was also less (46.23%) as compared to not needing it. This can be another benefit of antibiotics in acute exacerbation of COPD which has not been clearly stated in previous studies. The total median duration of hospital stay was six days which was consistent with other reported studies.^13^

There were certain limitations in our study. It was a single centre study with a non-probability convenience sampling and a smaller sample size. So, all of our findings may not be generalised. Next, we only studied the group of antibiotics used in our setting; not the individual antibiotics. Likewise, we did not include the reports of the microbiological study of sputum like other past studies. This could have provided a much better sense behind using a particular type of antibiotics.

## CONCLUSIONS

The use of antibiotics in acute exacerbation of chronic obstructive pulmonary disease was higher as compared to other similar studies. Out of them, only half of the cases fulfilled GOLD-2021 guidelines for antibiotics use. Penicillin was the most commonly prescribed group of antibiotics. So, there is a need for greater adherence to the standard guidelines regarding antibiotics use in COPD exacerbation.
